# Development of Two Analytical Methods Based on Reverse Phase Chromatographic and SDS-PAGE Gel for Assessment of Deglycosylation Yield in *N*-Glycan Mapping

**DOI:** 10.1155/2018/3909674

**Published:** 2018-03-29

**Authors:** Anahita D. Eckard, David R. Dupont, Johnie K. Young

**Affiliations:** Thermo Fisher Scientific, 180 Oyster Point Blvd, South San Francisco, CA 94080, USA

## Abstract

*N*-lined glycosylation is one of the critical quality attributes (CQA) for biotherapeutics impacting the safety and activity of drug product. Changes in pattern and level of glycosylation can significantly alter the intrinsic properties of the product and, therefore, have to be monitored throughout its lifecycle. Therefore fast, precise, and unbiased *N*-glycan mapping assay is desired. To ensure these qualities, using analytical methods that evaluate completeness of deglycosylation is necessary. For quantification of deglycosylation yield, methods such as reduced liquid chromatography–mass spectrometry (LC-MS) and reduced capillary gel electrophoresis (CGE) have been commonly used. Here we present development of two additional methods to evaluate deglycosylation yield: one based on LC using reverse phase (RP) column and one based on reduced sodium dodecyl sulphate-polyacrylamide gel electrophoresis (SDS-PAGE gel) with offline software (GelAnalyzer). With the advent of rapid deglycosylation workflows in the market for* N*-glycan profiling replacing overnight incubation, we have aimed to quantify the level of deglycosylation in a selected rapid deglycosylation workflow. Our results have shown well resolved peaks of glycosylated and deglycosylated protein species with RP-LC method allowing simple quantification of deglycosylation yield of protein with high confidence. Additionally a good correlation, ≥0.94, was found between deglycosylation yields estimated by RP-LC method and that of reduced SDS-PAGE gel method with offline software. Evaluation of rapid deglycosylation protocol from GlycanAssure™ HyPerformance assay kit performed on fetuin and RNase B has shown complete deglycosylation within the recommended protocol time when evaluated with these techniques. Using this kit,* N*-glycans from NIST mAb were prepared in 1.4 hr and analyzed by hydrophilic interaction chromatography (HILIC) ultrahigh performance LC (UHPLC) equipped with a fluorescence detector (FLD). 37 peaks were resolved with good resolution. Excellent sample preparation repeatability was found with relative standard deviation (RSD) of <5% for peaks with >0.5% relative area.

## 1. Introduction

Posttranslational modifications (PTMs) which expand and create diverse protein functions in most cases include the introduction of a well-defined functional group to amino acids such as acetate or phosphate moiety. However, separate from most PTMs, glycosylation is the enzymatic addition of different carbohydrate molecules in different structure to the peptide backbone of a protein. More than 50% of the proteins in human are glycoproteins. Glycosylation plays a crucial role in regulating or indicating key biological processes such as cell-cell signaling and protein folding [1, 2]. Two main types of glycosylation include* N*- and* O*-linked glycosylation.* N*-glycosylation is attached to Asparagine residue and the glycosylation site is identified from consensus sequence Asn*X*Ser or Asn*X*Thr in which *X* can be any amino acid other than proline [3]. Change in* N*-linked glycosylation can influence the glycoprotein stability, serum half-life, and efficacy (potency of glycoprotein), trigger immunogenicity, and impact antibody's ligand binding [4–7].

During process optimization, changes are made to process parameters to impact CQA of the biotherapeutics. Additionally during production and release of the drug product, any perturbation to the process such as changes in cell line, cell culture media, and other fermentation parameters can impose changes in CQA including* N*-linked glycosylation which in turn impact efficacy and safety of drug product. Therefore, consistency of these CQA's including glycosylation have to be monitored to maintain controlled function [8–11].

*N*-linked glycans generally contain a common core pentasaccharide (trimannosyl core sequence (Man3GlcNAc2)), and based on the composition of sugars in branches, they can be classified into 3 categories of high mannose, complex, and hybrid. The most common class of* N*-glycans attached to mAbs is the complex, biantennary type, while all classes have been detected to some level [12]. Fully humanized monoclonal antibodies contain typical* N*-linked glycan population being mainly sialylated and core-fucosylated; biantennary glycans contain 0, 1, and 2 terminal galactose as well as some level of high mannose and hybrid glycans. The sialylated glycans are at low abundance in recombinant IgG_1_ and IgG_2_ [13].

Typically* N*-glycans are released by action of chemical or enzyme (peptide* N*-glycosidase F (PNGase-F)), which cleaves selectively nonfucosylated and *α*-[1-6]-core-fucosylated* N*-glycans between Asn residue and GlcNAc of oligosaccharides. Enzymatic release involves a long incubation time of commonly 10–24 h at 37°C. Upon release of the glycans, in traditional practice, they are separated from protein backbone by chromatographic methods (solid phase extraction) such as reverse phase, ion exchange, or normal phase or nonchromatographic methods such as ultrafiltration, precipitation (with cold ethanol), or magnetic beads [14]. Then glycans are vacuum-dried for 2–4 hours and labeled with fluorescent dyes to enhance labeling efficiency and sensitivity for analysis with LC [15–17].

In summary, traditional* N*-glycan profiling sample preparation has taken between 1 and 3 days. However, with the advent of the new assays in the market, it has been demonstrated that* N*-glycan profiling results can be achieved at equal or better quality without going through lengthy workflows. In new workflows sample preparation time is a total of 30–90 min with rapid deglycosylation varying between 5 and 15 min, followed by labeling varying between 5 and 60 min and rapid clean ups taking 7–20 min.

Therefore the need to monitor* N*-glycan mapping for biotherapeutics during various phases of the biotherapeutic product's lifecycle such as clone selection, process optimization, and lot release testing [18–24] demands a more user friendly, short, and high throughput workflows that facilitate handling of large number of samples. When a suitable* N*-glycan mapping procedure is selected, it is important to evaluate and understand if current trend of rapid deglycosylation methods can reach completeness within the given time of the protocol. To quantify deglycosylation yield, analytical methods such as reduced CGE and LC-MS with online software have been used and reported as suitable methods with similar output [13, 14]. Also SDS-PAGE gel has been used as a nonquantitative gel-shift assay. Additionally various methods such as SEC, FTIR, DSC, and florescence spectroscopy have been evaluated but shown not to qualify as suitable techniques for quantification of glycosylation level. Therefore, in this work we have developed two new simple analytical methods of reverse phase chromatography and reduced SDS-PAGE gel assay with offline software (GelAnalyzer) for quantitative assessment of deglycosylation yield in* N*-glycan mapping workflow. Level of deglycosylation in samples processed with workflow from GlycanAssure HyPerformance APTS kit was evaluated with these two analytical methods, and the correlation of results from the two methods was demonstrated.

## 2. Materials and Methods

### 2.1. Materials and Reagents

SimplyBlue™ SafeStain from Novex by Life Technologies (Cat# LC6065), NuPAGE MOPS SDS running buffer (P/N# NP0001), NuPAGE LDS sample buffer (4x) (Cat# NP0008), NuPAGE Bis-Tris Mini Gels (Cat# IM-8042), GlycanAssure HyPerformance APTS kit (Cat# A33953), 1 M DTT (P/N# P2325), formic acid, 99.5+%, Optima™ LC/MS grade (P/N#A117-50) from Thermo Fisher Scientific, HPLC grade 100% acetonitrile (P/N#AC610010040) from Fisher Scientific, Aeris™ 3.6 *μ*m WIDEPORE XB-C18 (200 Å, LC Column 150 × 4.6 mm) (P/N 00F-4482-E0) from Phenomenex, Thermo Scientific™ Accucore™ 150 Amide HILIC LC Column (2.6 *μ*m, 150 A, 2.1 × 150 mm), and Thermo Scientific Vanquish™ Horizon UHPLC System, glycoproteins of RNase B and bovine fetuin from Sigma (P/N#J62334, lot R112C021) and QA-Bio (P/N# GCP 500 *μ*g, lot# B1B2-04), IgG1*κ* monoclonal antibody (NIST mAb #8671 (lot# 14HB-D-001)) in 12.5 mmol/L L-histidine, 12.5 mmol/L L-histidine HCl (pH 6.0) from NIST were used.

### 2.2. Sample Preparation for* N*-Glycans Release and Labeling with APTS

To evaluate the yield of rapid deglycosylation over the course of the reaction recommended by GlycanAssure HyPerformance protocol, a set of samples were prepared at 50 *μ*g protein input with reagents from this kit. In brief, in each 1.5 ml tube 13.3 *μ*l of HPLC water, 5 *μ*l of bovine fetuin (10 mg/ml), 6 *μ*l of denaturant, and 1.2 *μ*l of denaturation buffer were added, and the content was mixed with pipette and heated in thermomixer for 5 min at 80°C (open cap). Tubes were cooled for 2 min in room temperature; then 3 *μ*l of digestion buffer and 1.5 *μ*l of PNGase-F enzyme were added, mixed with pipette, and incubated at 50°C in incubator for 1, 3, 5, 8, and 10 min. For negative control, 5 *μ*l of glycoprotein, 22 *μ*l of HPLC water, and 3 *μ*l of digestion buffer were mixed in a tube (not heated). Immediately after deglycosylation 1 *μ*l of treated or negative control samples was prepared according to Section 2.3 for evaluation of deglycosylation yield with reduced SDS-PAGE gel assay.

Next to evaluating the quantification of deglycosylation yield for a range of protein inputs with reduced SDS-PAGE gel and RP-LC method, bovine fetuin and RNase B were denatured and enzymatically deglycosylated with reagents from GlycanAssure HyPerformance APTS kit. Samples were analyzed on both reduced SDS-PAGE gel assay and RP-LC (according to Sections 2.3 and 2.4). In brief, for 50, 80, and 100 *μ*g protein inputs 10, 16 *μ*l of 5 mg/ml, and 10 *μ*l of 10 mg/ml glycoprotein samples, respectively, were added to a microfuge tube containing 8.3 *μ*l (for 50 and 100 *μ*g input) and 2.3 *μ*l (for 100 *μ*g input) of HPLC water. For denaturation, 6 *μ*l of denaturant and 1.2 *μ*l of denaturation buffer were added to the tubes and mixed with pipette, and the reaction mixtures were heated at 80°C for 5 min. After cooling the tubes for 2 min at room temperature, 3 *μ*l of digestion buffer and 1.5 *μ*l PNGase-F were added and tubes were heated in incubator at 50°C for 10 min. For negative control all the deglycosylation reagents were replaced with HPLC water (reaction mixture was mixed but not heated).

To compare the performance of RP-LC method and reduced SDS-PAGE gel assay in quantification of deglycosylation yield, a set of samples were prepared with reagents from GlycanAssure HyPerformance kit. In brief, bovine fetuin at 80 *μ*g protein input (16 *μ*l of 5 mg/ml) was denatured at various temperatures of 60°C, 80°C, or 95°C for 5 min with 6 *μ*l of denaturant and at various concentrations of denaturation buffer varying from 0.15 to 2.4 *μ*l. After the tubes had cooled for 2 min in room temperature, 3 *μ*l of digestion buffer and 1.5 *μ*l of PNGase-F were added and tubes were incubated at 50°C for 10 min in incubator for enzymatic deglycosylation. Released glycans were prepared and analyzed with RP-LC method or reduced SDS-PAGE gel according to Section 2.3or 2.4, respectively.

Lastly, to evaluate the derivatized* N*-glycan mapping profile of samples prepared from IgG1*κ* monoclonal antibody (NIST mAb (#8671 (lot# 14HB-D-001)) with GlycanAssure HyPerformance APTS kit), glycoproteins were treated according to the assay kit protocol directed for 50 *μ*g protein input in 3 steps of* N*-glycans release, direct APTS labeling (no prior clean up), and final excess dye removal. Released labeled glycans were analyzed according to Section 2.5.

### 2.3. Reduced SDS-PAGE Gel Assay for Analysis of Deglycosylation Yield

To prepare samples for analysis, 2 *μ*l of reaction mixture containing released* N*-glycans (from 1.66 mg/ml protein) was mixed with 5 *μ*l loading buffer (30 *μ*l LDS buffer + 20 *μ*l 1 M DTT) and 3 *μ*l water and heated at 70°C for 10 min. 5 *μ*l of these samples were pipetted in each well of a 10% Bis-Tris Gels and run at 200 V for 40 minutes using 1x SDS running buffer (prepared by mixing 50 ml of 20x NuPAGE™ MOPS SDS running buffer with 950 ml of DI water). Afterward gels were washed with DI water and stained with SimplyBlue SafeStain (Novex® by Thermo Fisher Scientific). After staining for a few hours, the gel was destained in DI water. Quantification of deglycosylation yield was conducted with GelAnalyzer software.

### 2.4.  RP-LC Method for Analysis of Deglycosylation Yield

For quantification of deglycosylation yield with LC, Phenomenex Aeris WIDEPORE XB-C18 column at 3.6 *μ*m particle size and 150 × 4.5 mm (P/N#00F-4482-E0) was used at 80°C. For separation, samples were injected at 15 *μ*l and separated during 15-minute period at 1 ml/min flow rate at a gradient of mobile phase B from 15 to 40% in 15 min, 60% from 15.1 to 20 min, and 15% from 20.1 to 25 min, with mobile phase B being 0.08% TFA in acetonitrile and mobile phase A being 0.1% TFA in water.

### 2.5. UHPLC-HILIC for Analysis of* N*-Glycan Mapping

Labeled* N*-glycans were separated with Thermo Scientific Accucore 150 Amide HILIC LC Column (2.6 *μ*m, 150 A, 2.1 × 150 mm) at 50°C with Thermo Scientific Vanquish Horizon UHPLC System equipped with 2 *μ*l flow cell FLD at 455 nm and 500 nm excitation and emission wavelength, respectively, at high power lamp mode and 10 Hz of data collection rate. Separation was performed with 15 *μ*l injection volume at a 45 min gradient of B from 32% to 45%, 45.5–47 min at 60%, and 47.5–50 min at 32% at constant flow rate of 0.45 ml/min, with mobile phase B being 100 mM ammonium formate (pH 4.4) and mobile phase A being 100% acetonitrile.

## 3. Results and Discussion

Due to the high importance of monitoring unbiased mapping of* N*-linked oligosaccharides in protein therapeutics and advent of rapid deglycosylation reagents and methods, it is important to be able to evaluate and ensure complete deglycosylation is achieved using these protocols. Orthogonal analytical methods of reduced CGE and LC-MS have been the two widely used methods for evaluation of deglycosylation yield [13, 14]. In this work we have developed and demonstrated the suitability of two new analytical methods for this purpose; a RP-LC method and reduced SDS-PAGE gel assay with offline software (GelAnalyzer). Correlations of these two methods were evaluated in quantification of deglycosylation yield. Performance of* N*-glycan mapping of a selected rapid deglycosylation reagent kit and protocol in the market (GlycanAssure HyPerformance APTS) was evaluated with these analytical methods.

### 3.1. Reduced SDS-PAGE Gel Assay for Analysis of Deglycosylation Yield

**Figure 1:**
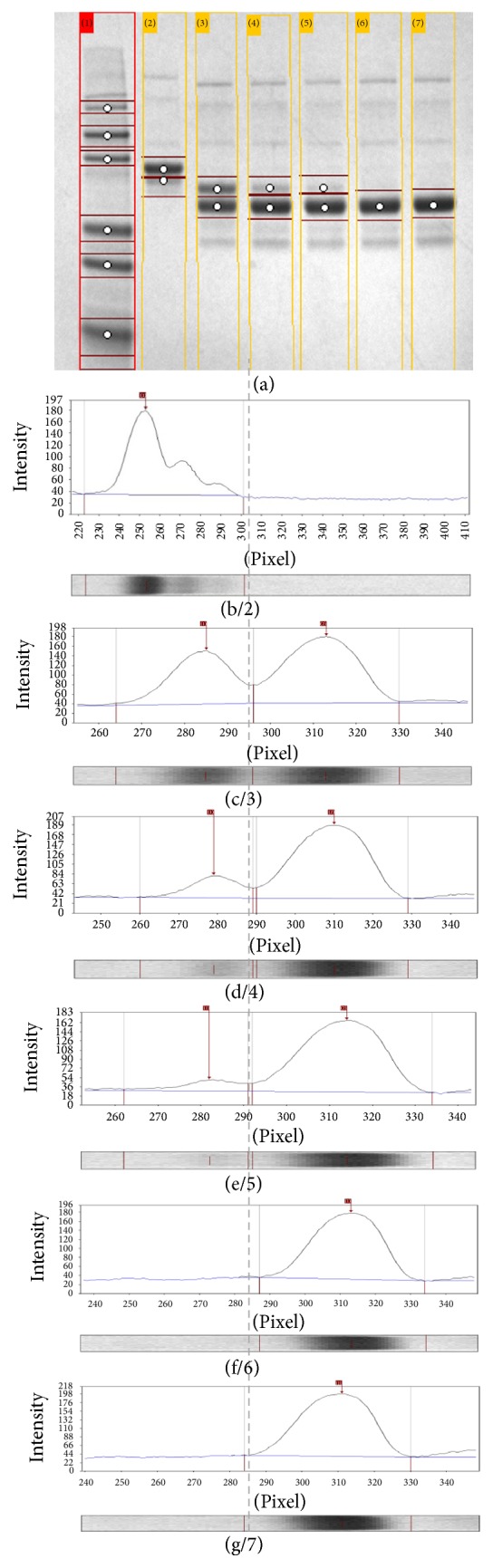
Method of quantification in level of deglycosylation with SDS-PAGE gel assay and GelAnalyzer software for a sample glycoprotein (fetuin). From left to right, numbers represent 1 (marker ladder), 2-b (glycosylated protein), 3-c (deglycosylated protein, 1 min), 4-d (deglycosylated, 3 min), 5-e (deglycosylated, 5 min), 6-f (deglycosylated, 8 min), and 7-g (deglycosylated, 10 min).

For application in* N*-glycan mapping assay to evaluate the performance of reduced SDS-PAGE gel method with offline software and its correlation with RP-LC method in quantification of deglycosylation yield of glycoproteins, a set of bovine fetuin samples were denatured and deglycosylated over a 10 min time course as described in Section 2.2, using reagents from GlycanAssure HyPerformance kit, and were analyzed on reduced SDS-PAGE gel according to Section 2.3. For analysis of data, a JPEG image of the gel was imported into GelAnalyzer software and processed according to user guidelines, in which the density of the bands in gel was converted to peaks (Figure 1(a)). And pixel volumes from each bands were used as area under the peak while glycosylated and deglycosylated peaks were integrated. Peaks that were not of interest for quantification such as enzyme peak were excluded from integration.

Peaks with lower molecular weight, deglycosylated protein migrated faster in the gel and appeared between pixel 290 and 335 on the peak profile, relative to the higher molecular weight glycosylated protein that appeared later in the gel between pixel 260 and 290 (Figures 1(b)–1(g)) on peak profile. In the case of the negative control sample, it was often observed that the glycosylated peaks migrated in two forms: one between pixel 220 and 260 and a lower molecular weight between 260 and 290. Therefore for negative control the raw volumes of these two bands were added together. Similar pattern was observed in RP-LC profile. Equation (1) was used to calculate the deglycosylation yield.

**Figure 2:**
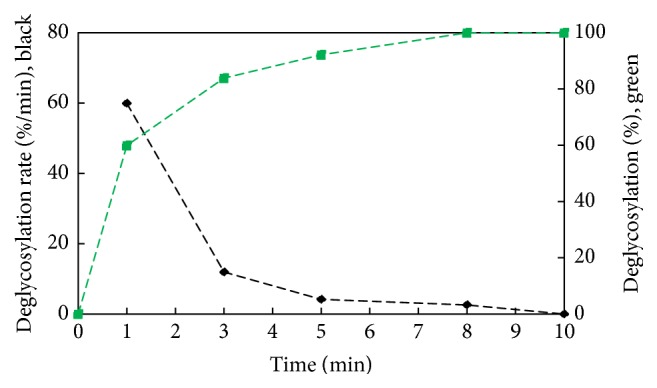
Enzymatic deglycosylation rate of bovine fetuin with GlycanAssure HyPerformance kit over the course of 10 min evaluated with reduced SDS-PAGE gel assay and GelAnalyzer software.

Data from deglycosylation time course study summarized in Table 1 were analyzed with aforementioned SDS-PAGE gel method. It was found that after 5 min denaturation of fetuin at 80°C with denaturant from GlycanAssure HyPerformance kit, 60% deglycosylation was obtained after 1 min, 84% after 3 min, 92% after 5 min, and 100% after 8 min (all samples were treated with PNGase-F for 10 min at 50°C). As can be observed in Figure 2, deglycosylation rate dropped rapidly from ~60% in the first min to 12% after 3 min, 4.2% after 5 min, and 2.6% after 8 min. This confirms that the enzymatic deglycosylation rate rapidly drops trough the course of the enzymatic deglycosylation reaction within the first 8 minutes reaching completeness after 10 min, thus eliminating the need for overnight incubation:

(1) %  deglycosylation=deglycosylated  peak  areaglycosylated  peak  area + deglycosylated  peak  area×100.

Previously reduced CGE with online software has been successfully shown by Cook et al., 2012, for quantification of deglycosylation yield of antibody samples at various incubation times with PNGase-F. In this method, heavy chain (HC) and light chain (LC) are migrated and resolved separately, and it is the ratio of peak area in degli HC relative to total area of gly HC plus degli HC that is measured for deglycosylation yield. While the two peaks of gly and degli HC are not often well resolved from each other, the resolution is acceptable for this application in antibody samples.

### 3.2. Quantifying Released Glycans with RP-LC Method

To demonstrate the quantification of deglycosylation yield with RP-LC method and compare that to results from SDS-PAGE, two glycoproteins of RNase B and bovine fetuin at 50, 80, and 100 *μ*g protein input were denatured and enzymatically deglycosylated with reagents from GlycanAssure HyPerformance kit according to Section 2.2. Samples were analyzed on both reduced SDS-PAGE gel assay and RP-LC (according to Section 2.4). It is anticipated that on a RP stationary phase, the glycosylated protein species were eluted first due to lower hydrophobicity followed by deglycosylated protein species that took longer due to higher hydrophobicity.

**Figure 3:**
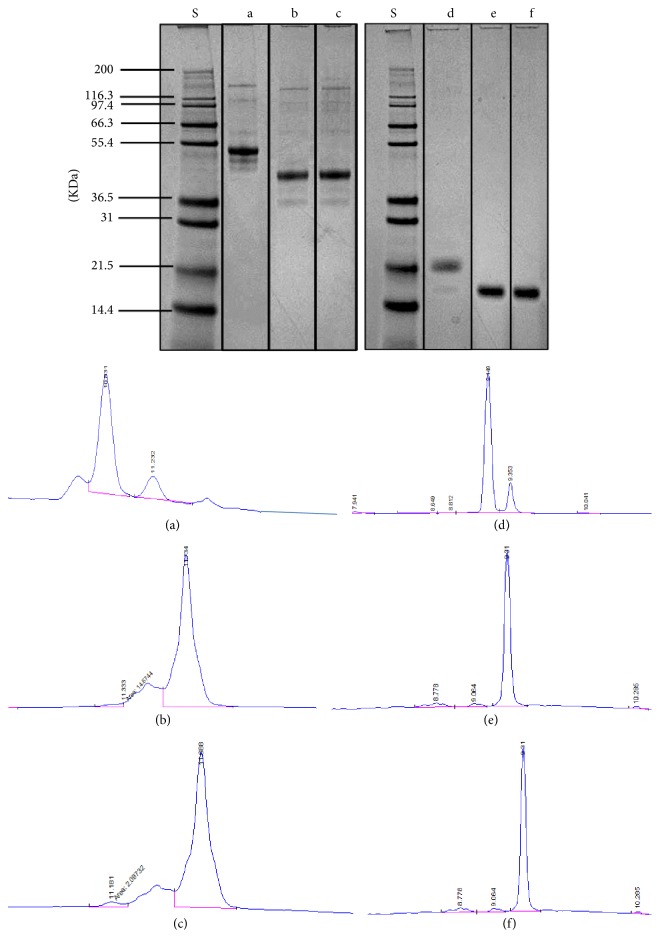
Quantification of level of deglycosylation in bovine fetuin (a–c) and RNase B (d–f) on reduced SDS-PAGE gel and RP-LC. Letter S represents Mark12 standard (2.5–200 kDa); a and d represent negative controls (glycoprotein sample solubilized in water replacing all deglycosylation reagents of PNGase-F enzyme, denaturant, and denaturation buffer); b and c represent deglycosylated bovine fetuin at 50 and 100 *μ*g input; and e and f represent deglycosylated RNase B at 50 and 100 *μ*g input.

As can be observed in Figures 3(a)–3(f), glycosylated fetuin peak was eluted at 11.1–11.3 min followed by a well resolved deglycosylated peak that was eluted at 11.6 min. Similarly for RNase B, glycosylated peak has been eluted at 9.1 min followed by a well resolved deglycosylated peak at 9.3 min. Similar to reduced SDS-PAGE gel assay, only these two peaks were integrated in the chromatograms, and quantification of deglycosylation yield was conducted based on (1). As observed in Figure 3, both gel and RP-LC data suggest that complete deglycosylation was achieved within 10 min period of the protocol from GlycanAssure HyPerformance assay kit for all protein inputs.

Previously, the extent of* N*-glycosylation site was determined and shown by Prien et al., 2015, with LC-MS tryptic peptide mapping followed by deglycosylation with PNGase-F. The percentage of occupancy of* N*-glycosylation site was determined from absolute peak areas of extracted ion chromatography (EIC) traces for glycosylated and aglycosylated peptides. Calculation with this method assumes that ionization efficiency of glycosylated and that of aglycosylated species are equal while in practice the ionization efficiency is likely to be higher in aglycosylated peptide containing Asn than deglycosylated peptide containing Asp residue. Therefore, the proportion of aglycosylated peptide is slightly overestimated [13]. Despite that, the results of deglycosylation yield on LC-MS method have been shown to match closely that estimated with CE-SDS method [13].

Zhang et al., 2011, have reported the use of various analytical methods to evaluate and understand the impact of deglycosylation on protein stability. Through evaluation of various analytical methods, it was found that intrinsic fluorescence spectra cannot point to difference between glycosylated and deglycosylated protein since they both have the same excitation and emission due to intact microenvironment around the Trp residues after PNGase-F digestion. Similarly dynamic light scattering (DSC) cannot be used as the thermal shift between glycosylated and deglycosylated protein is not significant. Similarly, size exclusion chromatography (SEC) was not able to show significant difference in profile between glycosylated and deglycosylated protein. Additionally, in Fourier transform infrared (FTIR) spectroscopy, the spectra from second derivative of Amide-I (without advance deconvolutions) did not indicate substantial changes in secondary structure of protein after in vitro deglycosylation.

Therefore methods of CGE, LC-MS, RP-LC, and SDS-PAGE with offline software are considered to be the most viable options so far for evaluation of glycosylation of glycoproteins, with the last two methods developed in this manuscript being the simplest methods among the four.

### 3.3. Correlation between RP-LC and Reduced SDS-PAGE Gel Methods

To compare the deglycosylation yield between the two analytical methods, an experimental design was conducted according to Section 2.2, in which bovine fetuin at 80 *μ*g protein input was treated in a full factorial experimental design at different denaturation temperatures of 60–95°C for 5 min and with different volumes of denaturation buffer (0.15–2.4 *μ*l) from GlycanAssure HyPerformance APTS kit. All treatments were enzymatically deglycosylated at equal condition for 10 min with 1.5 *μ*l of PNGase-F. Samples were analyzed on both reduced SDS-PAGE gel and RP-LC according to Sections 2.3 and 2.4 and results were processed according to Sections 3.1 and 3.2, respectively.

**Figure 4:**
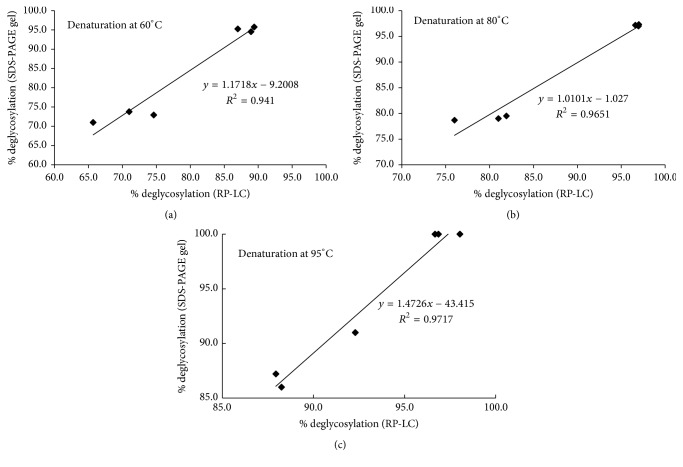
Correlation of deglycosylation yield of bovine fetuin with two analytical methods of reduced SDS-PAGE gel and RP-LC methods.

Correlations between deglycosylation yields quantified with two methods were plotted separately for each denaturation temperature. As can be observed in Figures 4(a)–4(c), a strong correlation coefficient was found between the deglycosylation yields obtained from two analytical methods: 0.94, 0.96, and 0.97 for 60°C, 80°C, and 95°C denaturation temperatures, respectively, which can be used both equally and interchangeably. These data also have shown that at a given concentration of denaturation buffer from GlycanAssure HyPerformance APTS kit, near 100% deglycosylation was obtained at temperatures as low as 60°C matching the yield with higher denaturation temperatures such as 80°C and 95°C.

### 3.4. Evaluation of APTS-Labeled Glycans on HILIC Column and UHPLC-FLD System

**Figure 5:**
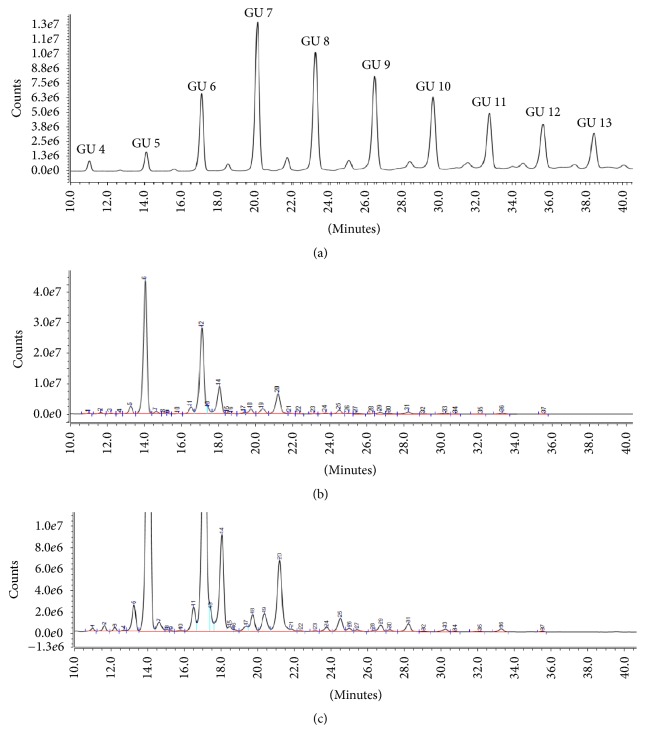
Chromatogram of dextran ladder (a),* N*-glycan profile of NIST mAb prepared with GlycanAssure HyPerformance APTS kit and analyzed on Accucore 150 Amide HILIC LC Column and Vanquish Horizon UHPLC equipped with FLD zoomed out (b) and zoomed in views (c).

**Figure 6:**
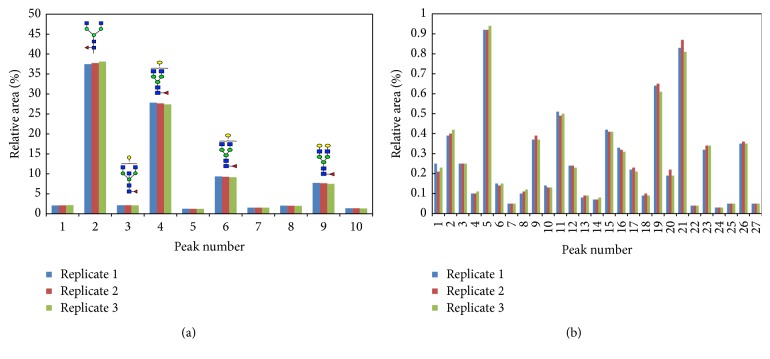
Relative area of three replicates of APTS-labeled* N*-glycans released from NIST mAb #8671 (lot# 14HB-D-001) grouped in two plots of peaks with relative area above 1% (a) and peaks with relative area below 1% (b).

*N*-glycans from NIST mAb were released with denaturation and enzymatic deglycosylation condition according to Section 2.2, labeled with APTS fluorophore tag, and cleaned with magnetic beads according to the protocol in GlycanAssure HyPerformance APTS assay kit. Along with the samples, dextran ladder, maltotriose, and maltotetraose were also labeled with APTS and analyzed. Only peaks between GU 4 and 13 were integrated as shown in Figure 5(a), which resulted in integration of 37 peaks in NIST mAb ≥0.03% relative area with good resolution. Excellent sample preparation repeatability (*n* = 3) with RSD of <5% was found for peaks with ≥0.5% relative area (Figure 6). Labeled glycans have shown a great sensitivity with limit of quantification (i.e., 0.1% relative area). Preparation of labeled* N*-glycan analysis took 1.4 hr with hands on time of <1 h.

## 4. Conclusion

Unbiased and fast mapping of* N*-linked oligosaccharides during various stages of a biotherapeutic product's lifecycle is necessary. Limited number of analytical methods have been reported to quantify the yield of deglycosylation achieved in a given* N*-glycan mapping assay. This is crucial for evaluation with the advent of rapid deglycosylation workflows in the market. We have demonstrated that RP-LC method can be used alone without MS, to evaluate deglycosylation yield due to well resolved glycosylated and deglycosylated proteins species separated based on their hydrophobicity. Similarly we have demonstrated that SDS-PAGE gel assay along with offline software (GelAnalyzer) can be used to calculate the yield of glycosylation. We have found that the results of deglycosylation yield quantified with the two methods are highly correlated with *R*^2^ of ≥0.94 and that they can be used interchangeably. Additionally we have used these methods to evaluate the performance of rapid deglycosylation technique in GlycanAssure HyPerformance APTS kit. Using these two methods, evaluation of rapid deglycosylation protocol from GlycanAssure HyPerformance kit has shown that complete deglycosylation is achieved within the recommended protocol time (15 min) for highly sialylated and high mannose glycoproteins of bovine fetuin and RNase B, respectively. Therefore, it was clearly demonstrated that traditional overnight incubation is not necessary. Lastly, we have evaluated the entire GlycanAssure HyPerformance kit for glycoprofiling of IgG1, NIST mAb on LC-FLD. While the sample preparation has taken <1.4 hr, we have found 37 peaks ≥0.03% relative area with great sensitivity and sample preparation repeatability with RSD of <5%.

## Figures and Tables

**Table 1:** Evaluation of enzymatic deglycosylation kinetics in fetuin processed with reagents and protocol from GlycanAssure HyPerformance APTS kit.

Sample lane # on the gel in Figure 1	2	3	4	5	6	7
Time of deglycosylation (min)	0	1	3	5	8	10
Glycosylated peak (raw volume^*∗*^)	3422	1912	686	275	0	0
Deglycosylated peak (raw volume^*∗*^)	0	2860	3560	3249	3482	3906
Deglycosylation (%)	0	60	84	92	100	100

^*∗*^Raw volume refers to density of each band from SDS-PAGE gel image representing the amount of glycosylated and deglycosylated proteins.
